# The relationships of nursing students’ satisfaction and self-confidence after a simulation-based course with their self-confidence while practicing on real patients in Vietnam

**DOI:** 10.3352/jeehp.2021.18.16

**Published:** 2021-07-30

**Authors:** Tran Thi Hoang Oanh, Nguyen Thi Yen Hoai, Pham Thi Thuy

**Affiliations:** Faculty of Nursing, Da Nang University of Medical Technology and Pharmacy, Da Nang, Vietnam; Hallym University, Korea

**Keywords:** Anxiety, Nursing students, Personal satisfaction, Self-concept, Vietnam

## Abstract

**Purpose:**

Simulation teaching refers to the replication of real-life scenarios, enabling students to practice nursing skills and learn actively in a safe environment. It also helps students control their anxiety and fears when caring for real patients. This study investigated the relationships of Vietnamese nursing students’ self-confidence in clinical practice with their satisfaction and self-confidence in simulation-based practice.

**Methods:**

This cross-sectional descriptive study included 182 nursing students. The data collection included 2 separate stages with 2 main questionnaires. The Student Satisfaction and Self‐Confidence in Learning Scale was used to measure students’ satisfaction and self‐confidence after learning in the simulation room. The Confidence Scale was used to measure students’ self-confidence when first performing techniques on actual patients. Data were analyzed by descriptive and Pearson correlation statistics.

**Results:**

Students’ satisfaction and self-confidence during the simulation course were quite high (mean±standard deviation [SD], 4.06±0.48 and 4.11±0.46 out of 5.0, respectively). In contrast, their confidence when first practicing on a patient was moderate (mean±SD, 3.19±0.62 out of 5.0). Students’ satisfaction showed moderate and weak positive correlations with self-confidence in pre-clinical practice and in clinical practice (r=0.33, P<0.001 and r=0.26, P<0.001, respectively).

**Conclusion:**

Simulation has become an effective teaching strategy that can help nursing students be well-prepared for clinical placements in Vietnam. An effective nursing education strategy is needed to enhance the satisfaction and self-confidence of nursing students in simulation and then in clinical practice to help achieve professional engagement and development.

## Introduction

### Background/rationale

Students’ satisfaction and self-confidence are the fundamental goals of nursing educators [1], especially students’ self-confidence when practicing on real patients. Satisfaction has been defined as a desire for delight or disappointment resulting from an event and a person’s prior expectations of himself of herself [2]. Self-confidence refers to a person’s belief that he or she can complete a task or achieve a desired goal [3]. In clinical nursing practice, self-confidence is an essential factor; it not only helps students complete their tasks accurately and precisely, but also build relationship and trust with their patients.

Simulations are one of the most interactive teaching and learning methods in nursing education. A simulation is defined as a situation or event created to be as similar as possible to clinical practice [4]. In simulations, students can apply theoretical knowledge to solve problems in various clinical-like scenarios and learn from errors in a safe and supportive environment, without harming patients. Simulations also allow students to practice nursing skills until they master them, providing them with an opportunity to improve their cognitive, affective, and psychomotor skills. Furthermore, simulations are the best method to help students overcome their nervousness and fear and to develop their self-confidence for real-life settings, thereby reducing medical risks and errors.

Some previous studies on the effectiveness of simulations in nursing education have shown moderately high results for improving students’ satisfaction and self-confidence in both pre-clinical and clinical settings [5-7]. However, few studies have focused on students’ self-confidence in clinical practice and the degree to which it is associated with their satisfaction and self-confidence in simulations.

### Objectives

The goal of the present study was to quantify the correlations of nursing students’ satisfaction and self-confidence during a simulation-based course with their self-confidence in performing procedures for the first time on actual patients.

## Methods

### Ethics statement

This study was approved by the Research Council of Danang University of Medical Technology and Pharmacy (no., 631/QD-DHKTYDDN). Informed consent was obtained from all participants.

### Study design

This was a single-group survey-based correlation study investigating participants’ psychosocial characteristics in different situations. It was described according to the STROBE (Strengthening the Reporting of Observational Studies in Epidemiology) statement (https://www.strobe-statement.org/).

### Setting

This study targeted second-year full-time nursing students in the Danang University of Medical Technology and Pharmacy from December 2018 to October 2019. In the fourth semester, nursing students were required to complete the fundamental nursing course, which included 2 parts: theory and preclinical practice. After the theory class, students participated in medium-fidelity simulations during preclinical practice, in which they could practice and role-play using static mannequins, anatomical representations such as cardiopulmonary resuscitation torsos, intravenous arms, and some high-tech mannequins with accessible pulses, breath, blood pressure, bowel sounds, and heart tone [8]. Upon finishing this course, they enrolled in the fundamental nursing clinical practice course, in which they had the opportunity to practice on a real patient under the supervision of a teacher or staff nurse.

### Participants

This study was conducted among 182 participants, who were selected from all second-year nursing students at Danang University of Medical Technology and Pharmacy, Vietnam who met the sampling criteria. Students who passed the theoretical part of fundamental nursing course were included in the study, while students who did not pass the simulation part of the course were excluded.

Students participated in a 2-step process. In step 1, after finishing the simulation part of the fundamental course, participants were asked to answer a demographic questionnaire and the Student Satisfaction and Self-Confidence in Learning tool. In the next step, after their first time providing care to a real patient, participants answered the Confidence Scale.

### Variables

There were 3 variables in this study: (1) students’ satisfaction with simulation-based learning; (2) students’ self-confidence after the simulation course (i.e., students’ belief in their ability to perform simulation activities and learn from them); and (3) students’ self-confidence in clinical practice (i.e., students’ belief in their own ability when first performing techniques on actual patients).

### Data sources/measurement

The Student Satisfaction and Self-Confidence in Learning tool (http://www.nln.org/professional-development-programs/research/tools-and-instruments/descriptions-of-available-instruments) was developed by the American Nursing Association based on Pamela Jeffries’ nursing education simulation framework. The tool includes 2 subscales that are used to assess the satisfaction and self-confidence of nursing students while using their knowledge to solve clinical situations with manikins. This instrument consists of 13 items, 5 of which measure satisfaction and 8 of which measure students’ confidence with the simulation teaching method after learning in the simulation rooms. Respondents answer items using a 5-point Likert scale, ranging from “strongly disagree” to “strongly agree,” with higher scores indicating higher levels of satisfaction and self-confidence [9].

The Confidence Scale was used to assess students’ self-confidence when practicing in the clinical setting. This scale was constructed and developed by Grundy [10] in 1993 to assess students’ level of self-confidence in performing caring techniques on actual patients in the hospital. It consists of 5 items, which are scored on a 5-point Likert scale from “not at all certain” to “absolutely certain” [10].

Both instruments were translated into Vietnamese using the forward-backward method and were tested for reliability in 30 trial samples (Supplements 1, 2). The Cronbach’s α values of the 2 subscales of the Student Satisfaction and Self-Confidence in Learning tool were 0.85 and 0.84, respectively. The Cronbach’s α of the Confidence Scale was 0.83. The Vietnamese versions of the 2 questionnaires were also sent to 5 nursing educators who had master or doctor of philosophy in nursing science degrees to assess their validity. The content validity index (CVI) of the satisfaction subscale and self-confidence in simulation subscale was 1.0 and 0.95, respectively. The CVI of the Confidence Scale was 1.0.

### Bias

In order to minimize potential sources of bias, researchers conducted the study on the entire relevant population to limit selection bias and tested the validity and reliability of the 2 questionnaires before use to reduce measurement bias.

### Study size

The sample size was calculated using G*Power ver. 3.1.9.4 (Heinrich-Heine-Universität Düsseldorf, Düsseldorf, Germany; http://www.gpower.hhu.de/). For the correlation test, the input was 1-tailed analysis, with an effect size of 0.2, an α error probability of 0.05, and a power of 0.8. A minimum sample size of 153 students was suggested. However, in order to enhance the generalizability of the study findings, the researchers included the entire relevant population. The actual sample size was 182.

### Statistical methods

Descriptive statistics were used to describe the characteristics of variables. Pearson correlation coefficients were calculated to assess the associations of nursing students’ satisfaction with simulation-based learning and their self-confidence during simulations and clinical practice. A P-value <0.05 was considered to indicate statistical significance.

## Results

### Participants

The participants ranged in age from 19 to 22 years old, with an average±standard deviation (SD) of 19.92±0.52 years. Most of the students were female (98.4%). The participants’ grade point average in their first year was moderate, with an average of 2.51±0.42.

### Nursing students’ satisfaction and confidence with simulation-based learning

Table 1 shows that the mean satisfaction of participants with simulation-based learning was quite high overall (mean±SD, 4.06±0.48 out of 5.0) (Dataset 1). The highest satisfaction score was for the teaching methods used in simulation. Most participants agreed that the methods were very helpful and effective (mean±SD, 4.21±0.62 out of 5.0), while they reported the least satisfaction for motivation from the teaching materials (mean±SD, 3.87±0.74 out of 5.0).

The self-confidence of students in simulation-based learning was moderately high (mean±SD, 4.11±4.06 out of 5.0). Most notably, the students felt self-confidence related to the materials that their instructors used for simulation education (mean±SD, 4.25±0.64 out of 5.0) and their responsibility to learn what they needed from the simulation (mean±SD, 4.24±0.57 out of 5.0).

### Self-confidence of nursing students when practicing in clinical situations

As shown in Table 2, students’ self-confidence when first performing practice care on real patients was moderately high (mean±SD, 3.19±0.62 out of 5.0). Of note, the students generally felt satisfied with the caring activity that they performed with real patients (mean±SD, 3.42±0.77 out of 5.0) (Dataset 1).

### Correlations of students’ satisfaction and self-confidence with the simulation in nursing education with their self-confidence in the clinical setting

Using the Pearson correlation test, students’ satisfaction and self-confidence in simulation-based learning showed positive correlations with their self-confidence in clinical performance (r=0.33 and r=0.26, P<0.001) (Table 3).

## Discussion

### Key results

Nursing students’ satisfaction with simulation-based learning was quite high. The highest score was found for satisfaction with the teaching methods used in the simulation classes. Students’ self-confidence in simulation and clinical practice was moderately high. Nursing students’ satisfaction and self-confidence in simulation-based learning showed significant positive relationships with their self-confidence in clinical practice.

### Interpretation

#### Nursing students’ satisfaction with the simulation classes

The highest satisfaction scores were found for the teaching methods used in pre-clinical practice lessons. Most students agreed that all of the teaching methods were effective and appropriate for students’ learning styles. Furthermore, students were enthusiastic about how the instructor taught the simulation lessons. Compared with traditional teaching strategies, simulation-based courses provide a positive learning environment with students at the center. Educators focus more on students’ strengths through valuable and exciting learning activities. These results are higher than those reported in a study of Winum [11] of satisfaction among nursing students.

Beyond the teaching methods, materials such as equipment, manikins, and medical consumable supplies played a pivotal role in affecting students’ learning, especially in simulations. This study showed that students were not completely satisfied with the practical materials. Specifically, some students still hesitated to agree that they were motivated and supported with adequate materials during the practical class. The result for this variable was not as high as the mean score of 4.21 reported by Zapko et al. [12]. This can be explained by the limited budget of the small university where this study was conducted, in which the nursing faculty lacks modern equipment and high-tech manikins, meaning that the simulation teaching method could not be applied to its full potential. Furthermore, students did not have the chance to use adequate consumable supplies. As a result of both insufficiencies, students were not able to practice as effectively as they desired, which may have affected their satisfaction.

Overall, the mean score for students’ satisfaction was fairly high (mean±SD, 4.06±0.48 out of 5.0). This result is quite similar to the finding of Lubbers and Rossman [13], who reported a mean score of 4.10±0.50.

#### Nursing students’ self-confidence in the simulation

The students generally agreed that the nursing educators effectively utilized the available resources to give the best possible simulation lessons. Similarly, with various active teaching and learning methods, students recognized their responsibility to learn what they needed from the simulation, and knew how to get support when they did not understand something during simulation practice.

The mean score for all participants’ self-confidence was 4.11±0.46 points out of 5.0. Thus, students’ self-confidence during the simulation was quite high, as this score is higher than was reported in a study conducted in the United States among nursing students (mean±SD, 4.00±0.46) [13].

#### Students’ self-confidence in clinical practice

Using the Confidence Scale to assess students’ level of confidence when first working with patients, the mean score was moderately high (mean±SD, 3.19±0.62). Students were generally certain that they performed correctly and did not hesitate when conducting their caring activities. A potential explanation for these results may be that the nursing students had the opportunity to practice freely in the simulation room before adapting to the clinical environment. It should also be kept in mind that they were not alone when they practiced with patients; instead, nursing lecturers or nursing supervisors were always next to them to observe and instruct them, which may have increased their confidence.

#### Correlations of satisfaction and self-confidence in the pre-clinical with self-confidence clinical environments

Satisfaction and self-confidence in the simulation practice showed positive correlations with self-confidence when students first practiced with patients (r=0.33 and r=0.26, respectively). The simulation teaching method is the best for creating a safe environment where students can have the opportunity to correct their mistakes while practicing nursing procedures. This is a valuable way to improve students’ self-confidence in clinical practice. In a study conducted among final-year nursing students in Australia, Meechan et al. [5] found that well-prepared integrated skills-based instruction in the university helped students acquire confidence before moving to the clinical setting.

### Limitations

The simulation and clinical environments are complex, with multiple confounding factors such as equipment resources, course arrangement, diversity of patients and the clinical environment, and differences in focus among various universities and hospitals. Therefore, this study’s results may not be applicable to other settings worldwide. Due to limited resources, this study focused on the satisfaction and self-confidence of nursing students. Future studies should explore other effects of simulation in nursing education.

### Generalizability

The researchers used the largest sample size possible by including the entire relevant population. Despite some confounding factors related to simulations and the clinical environment, the results of this study can be therefore applied to nursing education in Vietnam.

### Conclusion

Simulations have emerged as an effective teaching strategy that can help nursing students be well-prepared for clinical placements. This study found that nursing students’ satisfaction and self-confidence in simulation-based learning were correlated with their self-confidence in clinical practice. These findings furnish valuable evidence suggesting that nursing educators should strive to improve the quality of simulations to enhance students’ satisfaction and self-confidence in simulations and then in clinical practice. Such efforts may promote patient safety, quality of care, and professional engagement and development.

## Figures and Tables

**Table 1.** Participants’ satisfaction and confidence of participants with simulation-based learning

Variable	Mean±SD
Satisfaction with simulation-based learning	
1. The teaching methods used in this simulation were helpful and effective.	4.21±0.62
2. The simulation provided me with a variety of learning materials and activities to promote my learning of the medical-surgical curriculum.	4.11±0.56
3. I enjoyed how my instructor taught the simulation.	4.15±0.61
4. The teaching materials used in this simulation were motivating and helped me to learn.	3.87±0.74
5. The way my instructor(s) taught the simulation was suitable to the way I learn.	3.96±0.63
Average	4.06±0.48
Self-confidence of participants with simulation-based learning	
6. I am confident that I am mastering the content of the simulation activity that my instructors presented to me.	3.82±0.58
7. I am confident that this simulation covered critical content necessary for the mastery of the medical-surgical curriculum.	3.99±0.66
8. I am confident that I am developing the skills and obtaining the required knowledge from this simulation to perform necessary tasks in a clinical setting.	4.19±0.69
9. My instructors used helpful resources to teach the simulation.	4.25±0.64
10. It is my responsibility as the student to learn what I need to know from this simulation activity.	4.24±0.57
11. I know how to get help when I do not understand the concepts covered in the simulation.	4.19±0.66
12. I know how to use simulation activities to learn critical aspects of these skills.	4.06±0.67
13. It is the instructor's responsibility to tell me what I need to learn of the simulation activity content during class time.	4.16±0.67
Average	4.11±0.46

SD, standard deviation.

**Table 2.** Students’ confidence when practicing in a clinical setting

Variable	Mean±SD
I am certain that my performance is correct.	3.10±0.73
I feel that I perform the task without hesitation.	3.02±0.89
My performance would convince an observer that I’m competent at this task.	3.11±0.82
I feel sure of myself as I perform the task.	3.29±0.83
I feel satisfied with my performance.	3.42±0.77
Average	3.19±0.62

SD, standard deviation.

**Table 3.** Correlations of nursing students’ satisfaction and self-confidence during the simulation-based course with their self-confidence in clinical practice

Variables	Self-confidence in clinical practice	
	r	P-value
Satisfaction with simulation-based learning	0.33	0.001
Self-confidence with simulation-based learning	0.26	0.001

## Footnotes

**Data availability**
Data files are available from Harvard Dataverse: https://doi.org/10.7910/DVN/16AUXV Dataset 1. Nursing students’ response to the Student Satisfaction and Self-Confidence in Learning tool.

Dataset 2. Nursing students’ response to the Confidence Scale.

## Supplementary materials

Data files are available from Harvard Dataverse: https://doi.org/10.7910/DVN/16AUXV

Supplement 1. Vietnamese version of the Student Satisfaction and Self-Confidence in Learning tool.

Supplement 2. Vietnamese version of the Confidence Scale.

Supplement 3. Audio recording of the abstract.
