# Supplementary material for: The relationships of nursing students’ satisfaction and self-confidence after a simulation-based course with their self-confidence while practicing on real patients in Vietnam
Source: J Educ Eval Health Prof. 2021 Jul 30;18:16. doi: 10.3352/jeehp.2021.18.16 (PMC8382883; doi:10.3352/jeehp.2021.18.16)
Supplement: Supplementary file 3 — Supplement 1. Vietnamese version of the Student Satisfaction and Self-Confidence in Learning tool. [file jeehp-18-16-suppl1.docx]

Bộ câu hỏi

**SỰ HÀI LÒNG VÀ TỰ TIN CỦA SINH VIÊN KHI HỌC THỰC HÀNH**

**(Student Satisfaction and Self-Confidence in Learning)**

Anh (chị) vui lòng trả lời các câu hỏi dưới đây. Không có câu trả lời nào là đúng hay sai, anh (chị) đưa ra câu trả lời là đồng ý hoặc không đồng ý. Xin vui lòng đọc các mực độ đánh giá dưới đây trước khi anh (chị) trả lời câu hỏi.

1 – Hoàn toàn không đồng ý

2 – Không đồng ý

3 – Lưỡng lự (có thể đồng ý hoặc không)

4 – Đồng ý

5 – Hoàn toàn đồng ý

NA – Không có ý kiến

*Anh (chị) hãy đánh dấu “X” vào đáp án mà anh/chị chọn vào ô trống bên dưới.*

| **TT** | **Nội dung** | **1** | **2** | **3** | **4** | **5** | **NA** |
| --- | --- | --- | --- | --- | --- | --- | --- |
| **A. Hài lòng với phương pháp giảng dạy hiện tại** | | | | | | | |
| 1 | Phương pháp giảng dạy sử dụng trong bài thực hành có tác dụng và hiệu quả |  |  |  |  |  |  |
| 2 | Phòng thực hành cung cấp cho SV các dụng cụ và hoạt động thực hành đa dạng để giúp cải thiện khả năng thực hành của SV |  |  |  |  |  |  |
| 3 | SV hứng thú với GV giảng dạy thực hành |  |  |  |  |  |  |
| 4 | Dụng cụ giảng dạy tại phòng thực hành hỗ trợ và thúc đẩy khả năng thực hành của SV |  |  |  |  |  |  |
| 5 | Phương pháp giảng dạy của GV tại phòng thực hành phù hợp với phương pháp học của SV |  |  |  |  |  |  |
| **B. Sự tự tin của khi học thực hành** | | | | | | |  |
| 6 | Tôi tự tin rằng tôi hiểu rõ nội dung của các hoạt động thực hành do GV giảng dạy và hướng dẫn |  |  |  |  |  |  |
| 7 | Tôi tự tin rằng nội dung học thực hành bao trùm các nội dung quan trọng và cần thiết cho chương trình học của tôi. |  |  |  |  |  |  |
| 8 | Tôi tự tin rằng tôi đang phát triển dần các kỹ năng thực hành của mình và đạt được các yêu cầu về kiến thức từ việc thực hiện các kỹ thuật cần thiết tại phòng thực hành. |  |  |  |  |  |  |
| 9 | GV sử dụng các nguồn lực một cách hiệu quả để giảng dạy tại phòng thực hành. |  |  |  |  |  |  |
| 10 | Nhiệm vụ của SV là học những kỹ năng cần thiết từ các hoạt động thực hành tại phòng thực hành. |  |  |  |  |  |  |
| 11 | Tôi biết tìm kiếm sự trợ giúp khi tôi không hiểu nội dung thực hành tại phòng thực hành. |  |  |  |  |  |  |
| 12 | Tôi biết làm thế nào để dùng các hoạt động thực hành để tìm hiểu những kiến thức quan trọng của các kỹ thuật điều dưỡng. |  |  |  |  |  |  |
| 13 | Trách nhiệm của GV là giảng dạy và hướng dẫn cho tôi những nội dung tôi cần thực hành tại phòng thực hành. |  |  |  |  |  |  |
