# Supplementary material for: The relationships of nursing students’ satisfaction and self-confidence after a simulation-based course with their self-confidence while practicing on real patients in Vietnam
Source: J Educ Eval Health Prof. 2021 Jul 30;18:16. doi: 10.3352/jeehp.2021.18.16 (PMC8382883; doi:10.3352/jeehp.2021.18.16)
Supplement: Supplementary file 4 — Supplement 2. Vietnamese version of the Confidence Scale. [file jeehp-18-16-suppl2.docx]

Bộ câu hỏi

**THANG ĐÁNH GIÁ SỰ TỰ TIN KHI THỰC HÀNH LÂM SÀNG**

**(The Confidence scale)**

Khoanh tròn vào chữ số mô tả tốt nhất về khả năng hiện tại của bạn trong quá trình thực hành các kỹ thuật chăm sóc trên người bệnh tại bệnh viện (Lưu ý: mỗi câu hỏi chỉ chọn một đáp án phù hợp nhất)

**Câu 1: Tôi chắc chắn rằng các kỹ thuật tôi thực hiện trên người bệnh là chính xác.**

1 – Hầu như không chắc chắn

2 – Chỉ chắc chắn một vài bước

3 – Khá chắc chắn với những bước tôi làm tốt

4 – Chắc chắn với hầu hết các bước

5 – Hoàn toàn chắc chắn trong tất cả các bước

**Câu 2: Tôi nhận thấy rằng tôi không hề do dự khi thực hiện các kỹ thuật trên người bệnh.**

1 – Tôi thấy rất do dự

2 – Tôi có một chút do dự trong khi thực hiện

3 – Một số công việc tôi không thấy do dự

4 – Hầu hết các công việc tôi không thấy do dự khi thực hiện

5 – Hoàn toàn không do dự

**Câu 3: Việc thực hiện các kỹ thuật của tôi đã thuyết phục được GVHD/Điều dưỡng rằng tôi có năng lực thực hiện các kỹ thuật đó.**

1 – Hầu như không

2 – Đồng ý, nhưng chỉ một phần

3 – Một số trường hợp

4 – Hầu hết các trường hợp

5 – Toàn bộ các trường hợp

**Câu 4: Tôi cảm thấy chắc chắn về bản thân khi thực hiện các công việc trên người bệnh.**

1 – Hầu như không chắc chắn

2 – Đồng ý, nhưng chỉ một số ít lần

3 – Một số trường hợp

4 – Hầu hết các trường hợp

5 – Toàn bộ các trường hợp

**Câu 5: Tôi cảm thấy hài lòng về cách thực hiện các kỹ thuật chăm sóc trên người bệnh của tôi.**

1 – Không hài lòng

2 – Hài lòng rất ít

3 – Hài lòng trong một vài trường hợp

4 – Hầu hết các trường hợp

5 – Hoàn toàn hài lòng trong tất cả các trường hợp
